# Priority Actions and Progress to Substantially and Sustainably Reduce the Mortality, Morbidity and Socioeconomic Burden of Tropical Snakebite

**DOI:** 10.3390/toxins8120351

**Published:** 2016-11-24

**Authors:** Robert A. Harrison, José María Gutiérrez

**Affiliations:** 1 Alistair Reid Venom Research Unit, Liverpool School of Tropical, Liverpool L35QA, UK; 2 Instituto Clodomiro Picado, Facultad de Microbiología, Universidad de Costa Rica, San José 11501-2060, Costa Rica

**Keywords:** snakebite, envenoming, neglected tropical disease, antivenoms, prevention, global snakebite initiative

## Abstract

The deliberations and conclusions of a Hinxton Retreat convened in September 2015, entitled “Mechanisms to reverse the public health neglect of snakebite victims” are reported. The participants recommended that the following priority actions be included in strategies to reduce the global impact of snake envenoming: (a) collection of accurate global snakebite incidence, mortality and morbidity data to underpin advocacy efforts and help design public health campaigns; (b) promotion of (i) public education prevention campaigns; (ii) transport systems to improve access to hospitals and (iii) establishment of regional antivenom-efficacy testing facilities to ensure antivenoms’ effectiveness and safety; (c) exploration of funding models for investment in the production of antivenoms to address deficiencies in some regions; (d) establishment of (i) programs for training in effective first aid, hospital management and post-treatment care of victims; (ii) a clinical network to generate treatment guidelines and (iii) a clinical trials system to improve the clinical management of snakebite; (e) development of (i) novel treatments of the systemic and local tissue-destructive effects of envenoming and (ii) affordable, simple, point-of-care snakebite diagnostic kits to improve the accuracy and rapidity of treatment; (f) devising and implementation of interventions to help the people and communities affected by physical and psychological sequelae of snakebite.

## 1. Introduction

Snakebite is a medical emergency that annually kills over 95,000 people residing in some of the world’s most disadvantaged subsistence farming communities, and leaves over 300,000 surviving victims with permanent physical disabilities, stigmatising disfigurements and chronic mental morbidity [1,2,3]. It is the rural impoverished African, Asian and Latin American communities [4], and particularly the most economically-important and educationally-vulnerable 10–30-year-olds that suffer disproportionally high rates of snakebite mortality and morbidity. Snakebite is therefore a significant cause of death, poor quality of life and contributes to the depressing cycle of poverty in rural tropical communities.

While there have been a number of efforts to address this issue by some individual governments, civil society groups and members of the snakebite research community, a more globally-coordinated, multi-faceted approach is vital to achieve significant, sustained progress to reduce the medical and socioeconomic burden of snakebite. Here, the outcomes of a Wellcome Trust-funded Hinxton Retreat organised by the authors in September 2015, entitled “Mechanisms to reverse the public health neglect of snakebite victims” are summarized. This 36 participant workshop was organized to combine the breadth of knowledge, opinion, and experience of key physicians, scientists and advocates working on tropical snakebite in Africa (Nigeria, Kenya, Senegal, Angola), Asia (India, Sri Lanka, Nepal, Bangladesh, Vietnam), Latin America (Costa Rica, Brazil, Colombia), Oceania (Australia and Papua New Guinea), and Europe (UK, the Netherlands, Spain and Switzerland) with the fiscal, political and advocacy power of civil society groups and funding agencies—Médecins Sans Frontières (MSF), Drugs for Neglected Diseases initiative (DNDi), Global Snakebite Initiative (GSI), Health Action International (HAI), AMREF Health Africa, the World Health Organization (WHO) and the Wellcome Trust.

The first session of the workshop was an overview of the diverse factors limiting access to effective healthcare for tropical snakebite victims. As summarised below, poverty and limited government investment in snakebite management were described as ever-present themes at each level that snakebite affects its victims.

### 1.1. Increased Risk of Snakebite

Poor subsistent farmers are at greatest risk of snakebite [4] because (i) their mud/wattle homes provide little effective barriers to the ingress of venomous snakes; (ii) in many communities, people typically sleep on the floor without any protection from snakes seeking prey and warmth; (iii) lack of mechanised agricultural equipment exposes them to snakebite when farming and herding livestock; and (iv) footwear that would prevent many snakebites are either unaffordable or too uncomfortable in the prevailing hot, humid conditions.

### 1.2. Lack of Rapid Transport to the Nearest Health Centre

Rural snakebite victims typically gain access to health centres many hours (6+) after the bite [5,6], which increases both the severity of pathology and complexity of treatment. The causes of this delay vary significantly but often include (i) remoteness and lack of road infrastructure; (ii) poor ambulance services; (iii) expense of transport, when it is available and (iv) cultural norms dictating that snakebite victims often first seek assistance of traditional healers—whose administrations are lengthy, often harmful and rarely effective; and, finally, (v) there is often a loss of confidence in local health facilities because of a frequent lack of affordable and effective antivenom, which regrettably reinforces the importance of traditional healers in these communities.

### 1.3. Inadequate Snakebite-Management Capability in Tropical Rural Health Infrastructures

With the exception of charity-managed hospitals, most rural, remote health centres are under-resourced in many tropical countries. Antivenom treatment requires hospitalisation for a minimum of 24 h. Thus, while these health centres or dispensaries are closest to snakebite victims, they are often unable to provide effective clinical care because they lack the required clinical staff, hospital beds, equipment, effective antivenom and training in snakebite management. Snakebite victims are therefore often referred to a more centralised hospital, invoking further delay of treatment.

### 1.4. Poor Access to Effective Therapy

The very limited availability of effective, affordable and safe antivenom in much of rural sub-Saharan Africa and Asia, and in some regions of Latin America, is the most important contributor to this tropical disease burden [7]. Antivenom is IgG purified from the blood of horses/sheep hyper-immunised with snake venom—the consequence of this manufacturing protocol is that antivenom (i) often requires cold chain transport and storage; (ii) efficacy is largely restricted to the snake species whose venoms were used in manufacture—complicating the physicians’ choice of effective therapy; (iii) dose-efficacy is sometimes poor (particularly for polyspecific antivenoms) [8] and the high amounts of equine IgG (1.5–15 g in 2–20 vials of an antivenom formulated at 750 mg IgG/vial) required to reverse pathology pose significant adverse effect risks; (iv) costs, especially in snakebite endemic regions, are often passed onto the victims: a curative dose of the most effective, safe sub-Saharan polyspecific antivenom often costs a prohibitive US$400–700; making polyspecific antivenom one of the most expensive tropical disease treatments [9].

These financial, clinical and logistic complexities of antivenom treatment and the lack of accurate epidemiological data perhaps explain the typically limited government investment in effective antivenom purchase and distribution to rural communities in greatest need. The lack of robust regulatory control of antivenom efficacy also leads to widespread availability of less expensive but weakly or ineffective antivenoms throughout sub-Saharan Africa—which exacerbate the problem.

### 1.5. Problematic and Costly Medical and Surgical Intervention

Snakebite victims may need to be hospitalised because (i) of severe neurological, haemorrhagic, coagulopathic, renal, cardiac, or myotoxic manifestations of envenoming; (ii) antivenom must be intravenously administered; (iii) cure is complicated to assess; (iv) the frequent need to observe and treat patients for antivenom-induced adverse effects; and (v) extensive tissue necrosis subsequent to envenoming by some species requires surgical debridement or amputation to prevent spread of life-threatening sepsis. Effective hospital management of snakebite therefore requires specific clinical and surgical training—medical education that is often lacking in these remote, under-resourced regions.

### 1.6. Inadequate Post-Treatment Care

Post-treatment care rarely exists but is greatly needed to manage the chronic psychological [10] and physical disability needs of surviving snakebite victims—estimated at 300,000 annually. It seems obvious that the type of prosthesis, physiotherapy and psychotherapy counselling and assistance provided by charity groups to landmine victims, for example, would also help snakebite victims to become, once again, active, contributing members of their communities.

### 1.7. Governmental and International Health Agency Neglect of Snakebite Victims

The historical categorisation of snakebite as an accident/injury rather than a tropical disease, the fact it cannot be eradicated, the lack of accurate data evidencing the medical and socioeconomic disease-burden, its complex and expensive treatment, and competing tropical health priorities have combined in the nearly universal public health neglect of snakebite victims by governments and International Health Agencies. Worldwide, snakebite kills approximately 270–340 people every day—one fifth the number of people that die from malaria. In India, snakebite kills half the number of people that die from HIV [11]. In Africa, snakebite annually kills three times the number of people that died in the recent Ebola epidemic. 

The tragedy is that snakebite victims, despite their high rates of death and suffering, do not benefit from the generous national and international investment provided to reduce the disease burden of malaria, HIV, lymphatic filariasis, schistosomiais, and several other tropical diseases such as Buruli ulcers, trypanosomiasis and leishmaniasis that pose a lower threat of death, physical disability and psychological morbidity than snake envenoming [12]. 

Snakebite is a legitimate Neglected Tropical Disease [4,13], which has been sidelined to the margins of most treatises advocating action to alleviate the disease burden of NTDs. This retreat sought to devise systems to galvanise actions that will reverse this national and international neglect of tropical snakebite victims.

## 2. Priority Actions Identified and Progress to Meet the Retreat Objectives

After the above overview of the snakebite burden in Africa, Asia and Latin America, the participants were next tasked with identifying key strategic, and achievable, interventions to:

(i) reduce snakebite incidence and increase access to effective health care;
(ii) improve the clinical management of snakebite before, during and after hospital care;
(iii) apply scientific innovation to devise novel, effective, affordable and safe snakebite therapies and rapid diagnosis;
(iv) implement an effective snakebite advocacy initiative.

The following sections describe the priority objectives identified during the workshop (collated from the oral and written comments collected from participants after each session), and the progress achieved since the retreat or prior exemplars of effective practice in these key areas.

### 2.1. Reduce Snakebite Incidence and Increase Access to Effective Health Care—Priority Objectives

#### 2.1.1. Acquisition of Accurate Snakebite Disease Burden Data

A clear need was identified for public health and social research programs to capture data evidencing the medical, social and economic impact of snakebite envenoming. The avenue to achieve this would be collaborative (with Ministries of Health (MoHs) and medical charities) community- and hospital-based surveys recording snakebite incidence, mortality and morbidity data (including post-hospital treatment outcomes) in formats that can be utilized by health economists to generate health impact assessments. This information, collected for nations or regions, was identified as a critical step to (i) assess local, national and regional therapeutic needs; (ii) establish baseline data for determining the medical, health economic and sociological impact of preventative interventions; and perhaps, most importantly, (iii) to provide governments with the data they need to implement or approach International Health Agencies and philanthropic donors to fund evidence-based intervention strategies.

The social research programs would benefit from inclusion of studies to better understand snake ecology and behaviors dictated by rural poverty, identify especially vulnerable groups, such as indigenous communities, and determine the impact of snakebite on these groups, together with understanding the social and cultural contexts in which snakebites occur and their implications for prevention and appropriate management.

##### Current Progress and Prior Exemplars of Effective Practice

Data from studies conducted in India [11,14], Bangladesh [15], Sri Lanka [16,17], Nigeria [12,18], Burkina Faso [19], Kenya [20] and Senegal [21], amongst others, provide evidence of an increasing capability of acquiring snakebite epidemiology data that need to be adopted and promoted more widely. The workshop was informed of hospital-based and community survey projects being undertaken or planned in several countries represented by the recently established African Society of Venimology and in Southeast Asia.

#### 2.1.2. Rapid Access to Effective Treatment

There was broad consensus that motorcycle transport has proved very effective in reducing snakebite deaths in some settings [22]—and should be trialed in other communities, if appropriate. It was considered necessary to design strategies on the basis of the specific local ambulatory, ecological, cultural, social and institutional features, and with the active involvement of local communities. Sustaining such programs will require inculcation into the regional ambulance service, and, where appropriate, partnering with other civil society groups (e.g., mother and newborn health groups) to expand rural ambulatory systems to respond to all local medical emergencies.

##### Current Progress and Prior Exemplars of Effective Practice

The workshop was informed of an emergency response system designed to meet the specific needs of Papua New Guinean snakebite victims that contained many valuable aspects that could be implemented globally. Since the retreat, the Kenya Snakebite Study Group established in 2015 has acquired funds to trial the Kenya Snakebite Emergency Response System (*KenSERS*) in Baringo County. This is based upon mobile telephone activation of specially-designed, inexpensive motorcycle ambulances to speed rural snakebite victims to hospitals equipped with appropriately trained clinical staff provided with effective antivenoms. Success of the *KenSERS* pilot project will provide an affordable ambulance template to improve snakebite victims acquiring rapid access to effective treatment throughout sub-Saharan Africa (s-SA), and globally.

#### 2.1.3. Education Campaigns

Local, context-specific, public awareness campaigns are required to educate local communities in simple, inexpensive ways to protect themselves (footwear, bed nets, home design, etc.) and avoid harmful practices that increase the likelihood of snakebite. These would require MoH involvement and ideally incorporate the cultural stature of local traditional healers, and, importantly, with other local civil society initiatives and be part of universal health coverage. Education campaigns at the community level need to respect the cultural backgrounds. It was considered important that the education campaigns include a strategy of approaching the media, with the aim of targeting governments, public health authorities, international health agencies and foundations, and other philanthropic donors and stakeholders to acquire the political support and funding for consolidating a long-term, sustainable strategy to reduce snakebite incidence, mortality and morbidity.

##### Current Progress and Prior Exemplars of Effective Practice

Significant efforts have been performed in several countries. For instance, in Costa Rica, an active community education program has been underway for several decades, directed to rural areas of high snakebite risk, including indigenous communities. Likewise, an ongoing project in Papua New Guinea involves snakebite prevention campaigns at the local community level. A successful project was developed in Nicaragua, with the support of the Pan-American Health Organization (PAHO) aimed at developing integrated actions between the MoH and local traditional healers.

### 2.2. Improve the Clinical Management of Snakebite before, during and after Hospital Care—Priority Objectives

#### 2.2.1. Coordination and Standardisation of Clinical Snakebite Activity

Clinical research groups urgently need to establish clinical trials networks for collecting data post-treatment—unified under an international coordinating body. The task for this body will include (i) identifying standard metrics and protocols for evaluation of antivenom effectiveness at all levels from phase 1 dose-finding through to Randomised Clinical Trials, to generate consensus on endpoint measurement and safety assessment; (ii) providing guidance on endpoints; (iii) agreeing upon the most valuable prospective observational data using standardised and specific case definitions; and (iv) promoting clinical research aimed at identifying poorly known clinical manifestations of envenoming by some snake species in various regions of the world.

It was also acknowledged that there exists an urgent need for systematic programs to teach basic aspects of diagnosis and clinical management of snakebite envenoming at university, hospital and local health center levels. This should include the publication and wide distribution of guidelines and algorithms for clinical management of envenoming. The use of new Information and Communication Technologies offers opportunities to reach health staff in remote settings of high snakebite incidence.

##### Current Progress and Prior Exemplars of Effective Practice

The freely available WHO guidelines for the clinical management of snakebite in Africa [8] and Asia [23] need regionally organized efforts to ensure distribution to all areas and hospitals dealing with snakebite. Likewise, several countries have issued national guidelines for the diagnosis and treatment of snake envenoming. The workshop identified that the University of Adelaide’s week-long “Clinical Toxinology Short Course” is a type of training module that could be delivered more globally. The Papua New Guinean emergency response system also includes a hospital management of snakebite training module. There are groups actively working at improving clinical management of snakebites in several countries in Latin America, Africa, Asia and Oceania, but there is a need to integrate these efforts on a regional basis. The use of the informatics platform Elluminate by PAHO has been implemented for medical training programs on snakebite envenoming in Latin America.

#### 2.2.2. Effective First Aid Protocols and Training

Research into the development of first aid methods that are practical, effective and affordable at the community level was identified as another priority.

##### Current Progress and Prior Exemplars of Effective Practice

The WHO snakebite-management guidelines also include effective first aid recommendations. The Papua New Guinean emergency response system includes specific training modules on effective first aid that could be valuably adapted for other regions. It is encouraging that recent research [24] indicates the possibility of developing novel, perhaps pharmacological, first aid systems—whose effectiveness will require clinical testing.

#### 2.2.3. Improve Evaluation of Antivenom Efficacy

The existence of some antivenoms in sub-Saharan Africa of known ineffectiveness [6,25,26] and others of unknown efficacy highlight the importance of regulatory control and independent assessment of antivenom efficacy.

##### Current Progress and Prior Exemplars of Effective Practice

The WHO took the opportunity of this workshop to publicly announce the launch of a prequalification scheme of antivenoms for sub-Saharan Africa [27], wherein manufacturers were invited to submit dossiers of their antivenom manufacturing and testing systems. The evaluation of the submitted dossiers against a set of published WHO prequalification acceptability criteria is currently underway, alongside a systematic literature review to update the WHO databases. The WHO is also designing a system for independent antivenom testing, using venom standards, to complete this process. This significantly progresses the formulation of a process to ensure that only appropriate, effective antivenoms are distributed in s-SA. In parallel, there is an urgent need to strengthen the capacities of local regulatory agencies to ensure a proper assessment of antivenoms being distributed in these regions, a task that could be achieved through WHO-led international workshops and seminars.

In addition to a sustained international cooperative effort in the preclinical testing of antivenom efficacy being performed in Latin America for many years (e.g., [28]), it was also reported to the workshop that several research groups have been/are performing preclinical efficacy assessment of several antivenoms distributed in sub-Saharan Africa. Examples include studies on the neutralization of venoms of the saw-scaled viper, *Echis ocellatus* from various regions [29,30,31] and of the black mamba, *Dendroaspis polylepis* [32]. It was agreed that the acknowledged usefulness of these independent research activities would be substantially enhanced by greater coordination and division of the work load to ensure continent-wide antivenom quality control (QC) testing. It was also agreed that establishment of an international network of antivenom-efficacy testing laboratories in West, East, Central and Southern Africa, as well as in other regions, was a priority objective and should be factored into the new WHO initiative. Several laboratories in various regions of the world have expressed willingness to participate in such an initiative.

#### 2.2.4. Improve Antivenom Availability

Workshop presentations highlighted that antivenom production globally consisted of over 45 manufacturers with variant output capacity, QC-testing facilities, government sponsorship, market (some international, but most national), and that the recent, highly-publicised market failure of the Sanofi Pasteur antivenom (Fav-Afrique) illustrated the fiscal challenges that some companies face. Regional strategies to significantly increase antivenom availability need to consider this manufacturing heterogeneity, and many of these laboratories need to upgrade their technological platforms, infrastructure, good manufacturing practices (GMPs) and staff training; some also require urgent re-analysis of the venom mixtures used for immunization. Concerted dialogue between manufacturers, governments and regional WHO regional offices is necessary to effect the changes needed to increase the quality and quantity of antivenoms manufactured by these laboratories. 

Technology transfer programs for antivenom production, from established laboratories to countries that do not produce antivenoms, but have the requisite technological capacity, were also considered an important objective.

The workshop predicted that antivenom–efficacy tests will provide evidence and identify regions with serious therapeutic deficiencies. A strategy needs to be urgently formulated to provide funds to existing manufacturers (with spare manufacturing capacity) to produce antivenoms to meet the immediate needs of these regions (see also Section 2.3.5).

##### Current Progress and Prior Exemplars of Effective Practice

The organization of regional networks of antivenom manufacturers developed in Latin America in the last decade [33] constitutes an effective strategy to improve antivenom quality that could be usefully adopted in other continents. The authors are aware of, and have been part of, projects that have resulted in the provision of new antivenoms for regions at particularly high risk for snakebites in Nigeria, Sri Lanka and Papua New Guinea, but, as stated above, a sustained and comprehensive global plan needs to be implemented urgently. Likewise, the diffusion and implementation of the WHO 2010 Guidelines for the Production, Control and Regulation of Snake Antivenom Immunoglobulins [34], currently being updated, have been promoted in various regions of the world.

#### 2.2.5. Improve Post-Hospital Management of Snakebite Victims

It was recognized that the long-term physical and psychological impact of snakebite in poor rural communities is another priority for the snakebite community, and requires research to (i) measure medical and socioeconomic impact and (ii) devise locally appropriate strategies to reduce this impact. It was also clear that implementing these measures requires expertise from medical charities assisting victims of other medical emergencies, such as landmine accidents, survivors of which suffer similar disabilities as snakebite victims. The attention to the psychological sequelae of snakebite victims is a highly neglected area that demands urgent research and attention.

##### Prior Exemplars of Effective Practice

The recent research describing the chronic psychological impact of snakebite in Sri Lanka [10,35] and Iran [36] need to be implemented in other regions of high risk to acquire a more global view of the mental health burden of snakebite, and complemented with analyses of the benefits of physiotherapy, psychotherapy and prosthetic counseling.

### 2.3. Scientific Innovation to Devise Novel Effective, Affordable and Safe Snakebite Therapies and Rapid Diagnosis—Priority Objectives

#### 2.3.1. Improvements in the Design of Venom Mixtures for Antivenom Production

The selection of venoms included in the mixture to immunize animals for antivenom production is often made on an empirical basis. The technologies of proteomics, together with information on the epidemiology of snakebites and the identification of the most relevant venom toxins to be neutralized [37], allow for a knowledge-based selection of the most appropriate venoms to be used in the immunization mixtures. This scientific and technological expertise can be harnessed to design/re-design antivenom manufacture to increase the polyspecific efficacy of antivenoms [38].

##### Current Progress and Prior Exemplars of Effective Practice

A vast volume of information has been generated in the past decade defining the protein composition of venoms of the most medically-important snakes [39], and on the cross-reactivity of antivenoms against venoms, as judged both by preclinical neutralization tests and by immunochemical analysis [40,41]. This information is now being used to assess the spectrum of efficacy of antivenoms and to establish the most adequate mixtures of venoms to be used in immunization schemes for the production of antivenoms. It has resulted already in some modifications in the design of venom mixtures for immunization (personal communication from antivenom manufacturers).

#### 2.3.2. Next Generation Therapy for the Systemic Effects of Envenoming

It was agreed that innovative science, allied with contemporary technical platforms, has strong potential to overcome the poor dose-efficacy (less than 10% of antivenom IgG is therapeutic [29] and high costs of conventional antivenom (treatment with Fav-Afrique antivenom incurred costs of $500–$700—contributing to its limited demand and market failure). The challenge is to develop therapies for systemic envenoming, which are polyspecifically effective over a large snake species range and at low doses, pose no adverse-effects risks and do not require a cold chain. Achieving these product development profile criteria should provide (i) a substantial market (economies of scale) for the new products that will attract commercial manufacturing incentives and (ii) products that could be safely used in the field by both clinicians and non-clinicians, which will significantly extend clinical utility beyond the current restraints of hospital-administered antivenom.

##### Current Progress and Prior Exemplars of Effective Practice

Publications on the challenges of antivenom treatment of snakebite [42,43,44,45] describe many options for science-driven improvement in the efficacy, affordability and safety of antivenom treatment. In this context, one unexpected benefit of the recent MSF-driven publicity over the market failure of the FavAfrique antivenom has been a surge of scientific interest from within and external to the snakebite community for research to improve treatment. This includes research examining new reagents (e.g., human monoclonal and scFv antibodies, camelid VHH and small molecules), selection platforms (e.g., display systems) and protocols (e.g., antivenomics) that are distinct from conventional antivenom production protocols. The recent collaborative efforts of new and well-established research groups in this field offer the promise of rapid progress towards the development of a new generation antivenoms within the next decade – clinically and societally-important initiatives that require research investment. These will also require extensive preclinical and clinical testing—underscoring the importance of establishing preclinical testing and clinical trials networks.

#### 2.3.3. Next Generation Therapy for the Local Tissue-Destructive Effects of Envenoming

The lack of medicinal interventions to treat the tissue-necrotic effects of snakebite was also identified as a priority, and the science challenge is to develop a very-rapidly effective (minutes within the bite), thermostable, inexpensive and polyspecifically effective therapy, which, ideally, could be applied in field conditions rapidly after the bite.

##### Current Progress and Prior Exemplars of Effective Practice

Encouraging results on small peptide enzyme-inhibitors [46], fucoidans [47] and venom-specific camelid VHH [44] suggest that additional research with this focus will yield results with significant impact. Several research groups are exploring already existing drugs, originally designed for other pathologies, to determine their effectiveness against snake venoms [48].

#### 2.3.4. Point-of-Care Diagnosis

In the absence of diagnostic tools, physicians’ treatment decisions rely upon the victim’s description of the snake (but distinguishing harmless or weakly-venomous snake species from life-threatening species is notoriously inaccurate) and signs of local and systemic envenoming, which may develop slowly and vary greatly (depending upon snake, amount of venom injected, injection site, etc.). There was broad agreement that the provision of Point-of-Care diagnosis (for use in the community and hospital) would significantly improve the accuracy and outcome of clinical management of snakebite victims and should be a research priority, particularly in some regions of the world where a ‘syndromic approach’ to diagnosis of envenoming is difficult. The development of an epidemiological diagnostic tool, designed to identify envenoming by snake species, was considered valuable but of a lower priority than the Point-of-Care clinical tool.

##### Current Progress and Prior Exemplars of Effective Practice

Recent research on molecular diagnosis of snake bite [49] provides evidence of a renewed interest in diagnosis—the challenge now is to develop kits that are affordable and sufficiently rapid to be useful as clinical Point-of-Care tools. The recent preclinical development of a lateral flow diagnostic assay for detecting envenoming by Indian cobras and Russell’s vipers [50] suggests that Point-of-Care snakebite diagnosis may become a reality in the near future.

#### 2.3.5. Investment in conventional antivenom production for sub-Saharan Africa is needed immediately

The consensus view was that, while all the above research concepts hold considerable therapeutic and diagnostic promise, practical implementation is years away. There is therefore a compelling and urgent need for national and international health agency support and investment in the production, efficacy-testing and delivery of conventionally-manufactured antivenoms to those regions in greatest need, particularly in sub-Saharan Africa.

### 2.4. Implementing Effective Snakebite Advocacy—Priority Objectives

#### 2.4.1. Implementing a Wide Range of Advocacy Strategies to Greatly Increase the Global Awareness of the Impact of Snakebite

The overarching objective of the meeting was the formulation of a plan to reduce snakebite death and disability, especially in tropical countries of high snakebite incidence. There was unanimous agreement that (i) the future success of the priority actions identified during the workshop required effective advocacy at each level (scientific, technological, clinical, and public health); and (ii) that the snakebite community’s attempts at advocacy urgently needs to be supplemented with advocacy professionals and organizations. A very positive and unique aspect of this workshop was the presence of key NGO representatives who contributed significantly to the discussions and to the development of future plans.

##### Current Progress and Prior Exemplars of Effective Practice

We report here that action was immediately taken after the retreat to scale-up the role and capability of the Global Snakebite Initiative (GSI); with Health Action International (HAI) being co-opted to run the GSI Secretariat and tasked with devising an effective advocacy strategy based around a ‘theory of change’ that is likely to include many of the same objectives discussed at this workshop. Furthermore, the GSI Board of Directors was expanded to include representatives of sub-Saharan Africa and Asia.

Another important progression was the initiative supported by GSI, HAI and MSF and sponsored by the government of Costa Rica and co-sponsored by the governments of Afghanistan, Angola, Bangladesh, Burkina Faso, Cameroon, Chad, Gabon, Guinea, Kenya, Nepal, Nigeria, Pakistan, Papua New Guinea, Philippines, Senegal, and Uganda to run a snakebite-focused Side Event at the World Health Assembly in Geneva, May 2016. This event, attended by 140 people from many countries, proved a powerful opportunity to raise awareness of the public health burden of snakebite, the plight of snakebite victims and a strategic plan to reverse this. The event attracted the attention of the media and many organizations, which now needs to be exploited to maintain this advocacy momentum. Encouraging the WHO to upgrade the profile of snakebite either within the Neglected Tropical Diseases or Essential Medicines departments is an essential prerequisite to gaining the support of other International Health Agencies and donors.

An independent film company, funded by the Lillian Lincoln Foundation, is preparing an hour-long documentary focusing on the plight of rural snakebite victims, and was invited to the retreat to conduct interviews and gain background information. A five-minute film extracted from this documentary was presented at the WHA Side Event and released online [51], which has received wide attention. It is expected that the full documentary, to be released early next year, will provide powerful imagery to support our global advocacy needs.

## 3. The Challenges Ahead

The considerable progress made in the past year reflects the value of collaboration with new partners with distinct skills, and illustrates the enthusiasm that exists at many levels to achieve the aims of the workshop. This will be vital because the priority objectives identified during the workshop are daunting in their diversity, geographic and technical scope and need for coordination—particularly as funds to implement these have yet to identified. Therefore, a multi-component, inter-sectorial and inter-disciplinary approach, involving stakeholders in many different realms, should be implemented. The workshop’s conclusion that s-SA should be the focus of pilot projects of some of these new initiatives seems sensible and meets an urgent need. The greatest challenge to making these actions sustainably successful will be coordination and acquiring the cooperation and support of national Ministries of Health, International Health Agencies and civil society groups.

There was considerable workshop discussion as to the optimal strategy to overcome this challenge, and the need for an organization to coordinate efforts at the global level was emphasized. GSI is the only snakebite-focused charitable organization with a global remit and, as such, received support from the workshop participants as the most appropriate agency to undertake this role. This represents a formidable commitment. As described above, GSI has responded to this challenge by forming effective collaborative relationships with organizations including the WHO, HAI, MSF and others, as well as with Permanent Delegations of several governments in Geneva. It is expected that GSI will expand its scope of activities, its collaborative network and its coordinating role in the near future. 

In parallel, independent initiatives of various sorts in different regions of the world and promoted by a variety of actors need to be supported and implemented at all levels of action (grass-root organizations, academic consortia, local health authorities, NGOs dealing with public health, health workers, and people working in the communication arena, among others). The combination of decentralized activities at many levels and GSI-coordinated initiatives is expected to catalyze an outburst of activities aimed at improving the management of snakebite victims to significantly reduce deaths and morbidity.

## 4. Conclusions

In conclusion, the Hinxton Retreat participants recommended that the following highly summarised, priority actions be included in this strategy to reduce the global impact of snakebite envenoming:

(a) Collection of accurate global snakebite incidence, mortality and morbidity data to underpin advocacy efforts and help design of effective public health strategies.
(b) Promotion of (i) public education campaigns aimed at reducing snakebite incidence; (ii) ambulatory/transport systems to improve access to hospital treatment; and (iii) establishing regional antivenom-efficacy testing facilities to ensure that antivenoms provided to the hospitals are effective and safe.
(c) Explore funding models for investment in the production of antivenoms to address existing deficiencies in some parts of the world.
(d) Establish (i) a global system for training in effective first aid, hospital management and post-treatment care of snakebite victims; (ii) a clinical network to generate treatment guidelines and metrics and (iii) a clinical trials system—to improve the clinical management of snakebite before, during and after hospital care.
(e) Develop (i) novel, polyspecifically-effective and safe treatments of the systemic and local tissue-destructive effects of envenoming and (ii) an affordable, low-technology, point-of-care snakebite diagnostic kit to improve the accuracy and rapidity of treatment.
(f) Devise and implement intervention strategies to help the people and communities affected by physical and psychological sequelae of snakebite in order to reduce the extent of social suffering caused by this disease.
